# Exploring *Thinopyrum* spp. Group 7 Chromosome Introgressions to Improve Durum Wheat Performance under Intense Daytime and Night-Time Heat Stress at Anthesis

**DOI:** 10.3390/plants13182605

**Published:** 2024-09-18

**Authors:** Gloria Giovenali, Maria Lia Di Romana, Alessandra Capoccioni, Vinicio Riccardi, Ljiljana Kuzmanović, Carla Ceoloni

**Affiliations:** Department of Agriculture and Forest Sciences (DAFNE), University of Tuscia, 01100 Viterbo, Italy; gloria.giovenali@unitus.it (G.G.); marialia.diromana@unitus.it (M.L.D.R.); alessandra.capoccioni@unitus.it (A.C.); riccardi98@live.it (V.R.)

**Keywords:** wild wheat relatives, heat stress, stress physiology, proline, water-soluble carbohydrates, grain number, grain weight, yield stability

## Abstract

Durum wheat (DW) is one of the major crops grown in the Mediterranean area, a climate-vulnerable region where the increase in day/night (d/n) temperature is severely threatening DW yield stability. In order to improve DW heat tolerance, the introgression of chromosomal segments derived from the wild gene pool is a promising strategy. Here, four DW-*Thinopyrum* spp. near-isogenic recombinant lines (NIRLs) were assessed for their physiological response and productive performance after intense heat stress (IH, 37/27 °C d/n) had been applied for 3 days at anthesis. The NIRLs included two primary types (R5, R112), carriers (+) of a differently sized *Th. ponticum* 7el1L segment on the DW 7AL arm, and two corresponding secondary types (R69-9/R5, R69-9/R112), possessing a *Th. elongatum* 7EL segment distally inserted into the 7el1L ones. Their response to the IH stress was compared to that of corresponding non-carrier sib lines (−) and the heat-tolerant cv. Margherita. Overall, the R112+, R69-9/R5+ and R69-9/R112+ NIRLs exhibited a tolerant behaviour towards the applied stress, standing out for the maintenance of leaf relative water content but also for the accumulation of proline and soluble sugars in the flag leaf and the preservation of photosynthetic efficiency. As a result, all the above three NIRLs (R112+ > R69-9/R5+ > R69-9/R112+) displayed good yield stability under the IH, also in comparison with cv. Margherita. R112+ particularly relied on the strength of spike fertility/grain number traits, while R69-9/R5+ benefited from efficient compensation by the grain weight increase. This work largely confirmed and further substantiated the value of exploiting the wild germplasm of *Thinopyrum* species as a useful source for the improvement of DW tolerance to even extreme abiotic stress conditions, such as the severe heat treatment throughout day- and night-time applied here.

## 1. Introduction

Durum wheat (DW), *Triticum durum* Desf. (2n = 4x = 28), with a global production of 32.9 million tonnes in 2022 (https://www.igc.int/en/default.aspx, accessed on 10 September 2023), is one of the most important crops worldwide and definitely in the Mediterranean area, which provides about 60% of total DW production [1,2]. Here, however, climate-change-related phenomena are predicted to become ever more intense in the near future [3], with increasingly detrimental effects on DW cultivation [4]. This scenario, combined with the continuous loss of climatically suitable areas [5], poses a major challenge to the adequate production of high-quality DW-based food in the Mediterranean basin [6]. In this area, DW is predominantly grown under rain-fed conditions, and hence, environmental and climatic factors, such as temperature increase and precipitation scarcity, are expected to progressively hinder its proper development and potential yield [7].

Although cultivated wheats, including DW and bread wheat (*T. aestivum* L., 2n = 6x = 42), are widely adapted to grow in many world areas, including heat-prone, heat stress occurrences, defined as episodes of high temperature lying outside of the range typically experienced by the plant, have major negative impacts on their development and final yield [8,9,10]. During their life cycle, some growth stages are more susceptible than others: high temperatures experienced by wheat during flowering (anthesis) and grain filling were found to be extremely detrimental to grain yield compared with stress occurrence at vegetative stages, causing a severe photosynthesis reduction and carbohydrate content remodulation [11,12]. Wheat’s optimum temperature around anthesis is 21 °C, and exposure to higher temperatures, particularly beyond 31 °C, negatively affects physiological events that are major determinants of reproductive success, including gametogenesis, pollen viability and germination, pollen tube growth on the stigma and finally proper fertilisation, altogether leading to a strong reduction in grain number and overall grain yield [9,12,13,14]. At the same time, grain weight is determined by the grain filling rate and its duration; since heat stress is known to increase the grain filling rate, it can reduce the duration of grain filling and eventually seed weight [15]. Thus, severe and prolonged exposure to elevated temperatures during the extremely heat-sensitive flowering period is expected to be detrimental to overall wheat vitality, altering the normal plant physiology by causing a photosynthetic decline and accelerated senescence and also affecting spike tissues and functions, ultimately reducing seed set and development [16,17]. Another relevant issue is that both historical temperature observations and model projections have predicted a more pronounced increase in night temperature (HN) compared to that at daytime (HD), the former having risen 1.4 times faster than the latter in recent decades [12,18]. A combination of HD and HN temperatures during flowering exacerbates the negative effects of HD temperature alone on physiological mechanisms, including carbon/energy balance, source–sink relationships and reactive oxygen species (ROS) production, and results in an enhanced reduction in seed set, grain number, grain weight, biomass and final grain yield [12,18,19,20,21,22]. All this evidence makes the improvement of heat tolerance at anthesis a priority in wheat breeding [16,23,24].

To counteract the stress factors, plants activate a dense response network, going from stress perception to cellular genome-wide reprogramming. The underlying mechanisms encompass short-term avoidance or acclimation strategies, mainly based on the control of water status and associated stomatal response, as well as long-term tolerance mechanisms, including a variety of protective systems such as ROS scavenging antioxidant systems and the accumulation of osmolytes [23,25,26]. The latter are small organic molecules that contribute to maintaining homeostasis and cell turgor, providing the driving gradient for water uptake and also removing excess levels of ROS, thus re-establishing the cellular redox balance upset by the stress [25,27,28]. Among the various osmolytes that accumulate in response to stress, high levels of soluble sugars and proline in target tissues were often associated with increased heat tolerance in wheat [29]. Although flag leaves, as the major photosynthetic organs in wheat at advanced growing stages, are the most widely studied in terms of osmolyte adjustment, a number of studies highlighted that spikes also represent major suppliers of photosynthates and contribute to enhanced tolerance to abiotic stress [30,31]. In particular, a higher proline accumulation in wheat spikes than in flag leaves was reported under heat- and water-limited conditions [32,33]. As for sugar content, El Habti et al. [34] reported that water-soluble carbohydrates (WSC) were predominantly allocated into the spikes of modern varieties, even if the combination of heat and drought stress did not further increase their amount, unlike observed in older genotypes.

To provide modern DW varieties with efficient stress-responsive mechanisms, the enlargement of the reduced wheat genetic basis via the exploitation of ample genetic resources of wild wheat relatives, naturally adapted to survive and reproduce in hostile climates and environments, offers good promise [35,36,37,38]. Besides more readily accessible species, like the direct progenitor of DW, i.e., the wild emmer *T. dicoccoides* (same AABB genome) and other *Triticum* and *Aegilops* spp. sharing one closely related genome [17,23,39], more distant relatives, such as those belonging to the *Thinopyrum* genus, are extremely interesting candidates. In fact, the genus includes several perennial species, native and naturally adapted to harsh environments of southern and eastern Europe, western Asia and northern Africa [40,41]. In recent years, a number of DW-*Thinopyrum* spp. recombinant lines have been developed through the “chromosome engineering” strategy [42,43]. This cytogenetic approach, consisting of the transfer of segments of alien chromosomes carrying desired genes to wheat chromosomes [44], allows incorporation into the crop genome of only small amounts of the wild chromatin, thus substantially reducing the occurrence of unwanted linkage drag. The DW-*Thinopyrum* spp. recombinant lines obtained, initially targeted for major disease resistance attributes [42,43,45,46,47], were subsequently field tested in several environments, including stressful ones, where they showed positive effects on yield-related traits associated with specific *Thinopyrum* spp. group 7 chromosome regions [48,49,50,51]. Recently, the great potential contribution of such lines to abiotic stress response was substantiated by experiments under controlled conditions, namely salt (NaCl) stress applied onto juvenile plants grown in hydroponics [52], as well as heat and combined heat+drought stresses applied to adult plants at the critical stage of anthesis [53].

**Figure 1:**
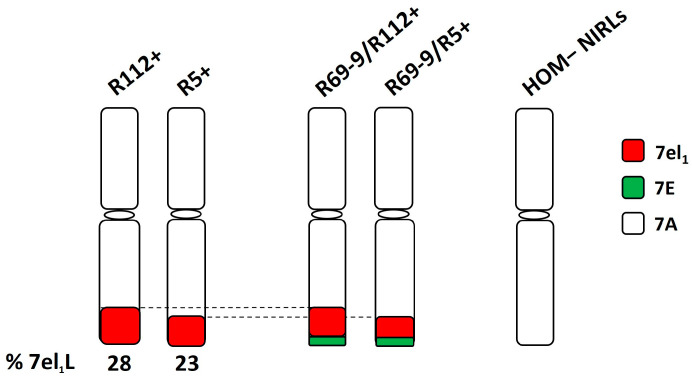
Durum wheat *Thinopyrum* spp. near-isogenic recombinant lines (NIRLs) tested under intense heat conditions. 7el_1_L: chromosome segment derived from the long (L) arm of the *Th. ponticum* 7el_1_ chromosome; 7E: donor chromosome of *Th. elongatum* “nested” segments into 7el_1_L; 7A: durum wheat recipient chromosome, present in NIRLs lacking any alien introgression (HOM−).

In the present work, the overall performance of DW-*Thinopyrum* spp. near-isogenic recombinant lines (NIRLs) was evaluated upon treatment with intense heat (IH) stress, consisting of both HD and HN temperatures, imposed on plants at anthesis. The stress conditions applied in the present experiment were more severe in extent and duration than in previous assays [53,54]. In particular, in the study of Giovenali et al. [53], in which the anthesis stage was similarly targeted, and the heat stress was likewise applied during 3 consecutive days, the treatment consisted of a progressive temperature increase from 22 °C up to 38 °C, maintaining this last temperature for 2 h, before proceeding to a gradual decrement from 38 °C to 22 °C (total treatment duration 6 h/day). In this heat treatment, two DW-*Th. ponticum* NIRLs, named R5+ and R112+ [42], stood out for their tolerant behaviour, as expressed by physiological and biochemical parameters, as well as by the remarkable stability of yield-related traits. Thus, one of the aims of the present work was to analyse the behaviour of the same two DW-*Thinopyrum ponticum* NIRLs, when subjected to more intense heat stress. A further aim was to test for the first time the IH stress response of more recently developed, composite DW-*Thinopyrum* spp. NIRLs, containing “nested” *Th. elongatum* introgressions into the same *Th. ponticum* segments of R5+ and R112+ [47]. Thanks to the different DW-*Th. ponticum* and *Th. ponticum*-*Th. elongatum* chromatin breakpoints present in the different recombinant chromosomes (see Figure 1) and to the availability of ideal control lines (near-isogenic lines without any alien introgression; see Section 2.1), the elucidation of the stress response mechanisms activated by the recombinant lines, also in terms of yielding ability/stability, could allow them to be tentatively associated with the presence of specific wild chromatin introgressions. To these aims, physiological and biochemical traits were monitored on plant materials grown under controlled conditions. Main yield parameters were also measured on stressed and control plants to identify the best-performing genotypes under an HD + HN temperature regime imposed during a critical stage for yield formation.

## 2. Materials and Methods

### 2.1. Plant Materials

In the present experiment, different DW-*Thinopyrum* spp. recombinant lines were employed as the main target genotypes (Figure 1).

They included two primary DW-*Th. ponticum* NIRLs, originally named R5-2-10 and R112-4 [42] and here referred to as R5+ and R112+, respectively. Such NIRLs were obtained through a chromosome engineering strategy [42] and developed in the background of the Italian DW cv. Simeto by five backcrosses followed by several self-generations. R5+ and R112+ possess 23% and 28% of the distal end of their recipient DW 7AL arm replaced by corresponding portions of the 7el_1_L arm of the tall wheatgrass species *Th. ponticum*, respectively. Besides them, two secondary NIRLs were also employed, named R69-9/R5+ and R69-9/R112+ derived from the primary types R5+ and R112+, respectively [47]. The latter NIRLs possess a *Th. elongatum* 7EL chromosome segment distally inserted (“nested”) into the respective 7el_1_L ones. The symbol “+” indicates homozygous carriers of the wild chromatin (HOM+), while the symbol “−” is used to indicate corresponding homozygous non-carrier sib lines (HOM−), also included in all analyses. Finally, the DW cv. Margherita, ICARDA-bred, was introduced as a positive tolerant control, since it expressed a remarkable heat tolerance in a range of stressful environments, including the Senegal River Basin, characterised by temperature extremes during most of the growth cycle [55,56].

### 2.2. Plant Growing Conditions and Experimental Design

**Figure 2:**
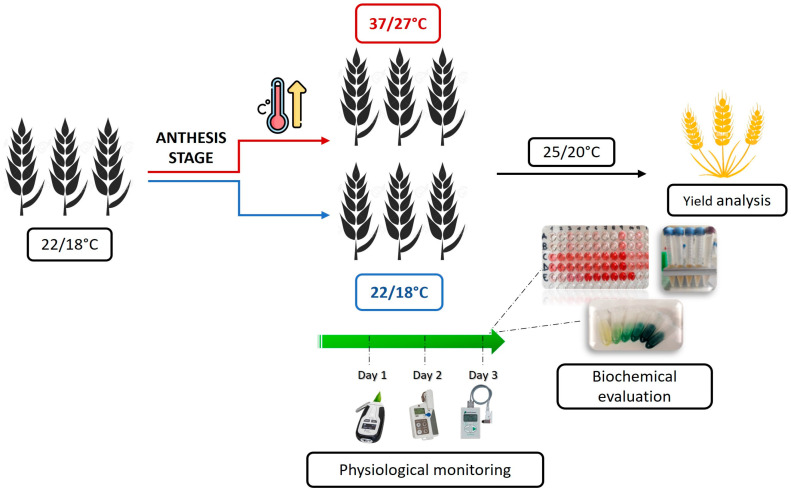
Experimental setting, modalities of stress application and data recording.

Plant materials were grown under controlled conditions in walk-in growth chambers (2.7 m L × 1.7 m W × 2.4 m H). About 270 total plants were grown and placed in the chamber in a randomised arrangement. This allowed for the standardisation of growth conditions and minimisation of environmental variability. Half of the total plants were maintained under control (C) conditions throughout the growth cycle, and the other half were subjected to an intense heat (IH) treatment, resulting in 15 replicate plants/genotype/treatment available to use for the various analyses, as specified below. Seeds were sterilised for 5 min in a sodium hypochlorite solution, rinsed twice and soaked for 2 h in distilled water. Sterilised seeds were germinated in Petri dishes on wet filter paper, and then one plant/pot was transplanted into 1.4 L pots containing soil, sand and perlite in a 6:1:1 ratio. The potted plantlets were maintained for about 2 weeks at 8/6 °C day/night (d/n) temperature and 12 h photoperiod to simulate the vernalisation phase. Plants were then grown at 16/12 °C and a 16 h photoperiod until tillering, at 20/16 °C until booting, at 22/18 °C until the early grain filling period and finally at 25/20 °C until full ripening. Relative humidity was maintained at around 60%. During the growth cycle, all plants, both the IH-stressed and the C group, were uniformly fertilised (1 g/L of Triabon, a slow-release fertilizer with N16-P8-K12-Mg4 plus trace elements) and well watered (monitoring performed every other day) to keep soil moisture close to field capacity, thus ensuring water was not a limiting factor. When plant main tillers reached the anthesis stage (Zadoks scale 61-6 5; [57]), plants to be subjected to the IH treatment were moved into an adjacent chamber set at 37/27 °C and maintained therein for 3 consecutive days, before being moved back to standard (C) growing conditions. Based on a literature survey (e.g., [34,58,59]), it was expected that the applied temperature regime would be effective in clearly discriminating tolerant vs. sensitive wheat genotypes to severe heat stress. The experimental setting, modalities of stress application and main analyses performed are illustrated in Figure 2.

Several physiological, biochemical and yield-related traits were assessed during and after the stress treatment as well as at maturity. They are listed in Table 1, together with their respective acronyms and measurement units, and the methods for their recording are described in the following paragraphs.

### 2.3. Physiological Traits

Chlorophyll content (SPAD), chlorophyll fluorescence (F_v_/F_m_, PI) and stomatal conductance (SC) were measured during the 1st, 2nd and 3rd day after anthesis on stressed and control plants. SPAD measurements were taken on 3 positions along the adaxial surface of the flag leaf (FL) using a hand-held chlorophyll meter (SPAD-502 Plus; Konica-Minolta, Hino-shi, Tokyo, Japan). The 3 figures were averaged to give a mean value for each plant and time point. To evaluate chlorophyll fluorescence, the OJIP curve (Fluorescence Transient) was recorded using a portable fluorometer (PAR-FluorPen FP110; PSI—Photon Systems Instruments, Drasov, Czech Republic) on FL adaxial surface after a dark adaptation of 30 min with detachable clips. The OJIP curve provides detailed information about the status and functionality of photo-system II (PSII) reaction centres (RC), allowing to extrapolate the F_v_/F_m_ (max photochemical efficiency of PSII) and Performance Index (PI) parameters. The latter is a combined measure of three factors: quantity of the photosynthetic RCs, maximum energy flow reaching the RCs and electron transport at the beginning of illumination. So, it expresses the energetic bifurcations of PSII. SC was measured in the middle portion of the FL adaxial surface using an SC-1 leaf porometer (METER Group, Inc., Pullman, WA, USA), previously calibrated into the growth chamber according to the company’s instructions. At least 10 plants for each genotype and treatment condition were used to assess the physiological parameters describing photosynthetic and stomatal performance.

Relative water content (RWC) determination was performed 3 days after anthesis on a 5 cm-long section cut from the middle portion of the fully expanded penultimate leaf of the main culm and calculated with the following formula: RWC = (FW − DW)/(TW − DW), where FW, DW and TW stand for fresh, dry and turgid leaf weight, respectively. At least 5 plants for each genotype and treatment condition were used to assess RWC.

### 2.4. Biochemical Assays

Proline (Pro) and water-soluble carbohydrates (WSC) were quantified on the FLs (Pro-FL, WSC-FL) and spikes (Pro-SP, WSC-SP) of stressed and control plants. FLs were also used to perform the lipid peroxidation assay. Tissue sampling was carried out 3 days after anthesis: FLs were collected in tubes, whereas spikes were wrapped in aluminium foils. Each tissue was immediately suspended in liquid nitrogen and stored at −80 °C until further use. Pro content, expressed as µmol/g FW, was measured following the ninhydrin-based colorimetric assay [60]. WSC content, expressed as g/g DW, was determined following the modified anthrone method reported by El Habti et al. [34]. Lipid peroxidation was evaluated by quantifying malondialdehyde (MDA) content (µmol/g FW) via the thiobarbituric acid reactive substances (TBARS) assay [61]. At least 3 plants for each genotype and treatment condition were used for Pro, WSC and MDA quantification.

### 2.5. Yield-Related Parameters and Stress Indices

All yield-related traits were measured when plants reached complete ripening and post-harvest. They consisted of traits assessed on the main (1st) tillers of each plant, i.e., those precisely targeted for IH stress at anthesis, and traits measured at the whole-plant level. Among derived traits, the spike fertility index (SFI) was calculated as the ratio between grain number per spike (GNS) and spike chaff weight (g), and the harvest index (HI) was calculated as the ratio between grain yield per plant (GYP, g) and total biomass (g). Measurements of all yield-related parameters were derived from at least 10 plants for each genotype and treatment combination.

The productive performance of all genotypes under heat-stressed vs. standard conditions was also comparatively evaluated on the basis of several stress indices, commonly used to assess the effect on grain yield (or even specific yield components, e.g., [24]) of abiotic stresses, mainly heat and drought, and hence to identify and select stress-tolerant genotypes [62,63,64,65]. The 9 indices tested here included the following: tolerance index (TOL), mean productivity (MP), geometric mean productivity (GMP), stress tolerance index (STI), harmonic mean (HM), stress susceptibility index (SSI), yield index (YI), yield stability index (YSI) and relative stress index (RSI). Microsoft Excel was used to calculate stress indices, using the following formulas:

Tolerance index (TOL) = Yp − Ys

Stress susceptibility index (SSI) = (Ys × Yp)/(Ȳp)^2^

Stress tolerance index (STI) = (Ys × Yp)/(Ȳp)^2^

Yield index (YI) = Ys/Ȳs

Yield stability index (YSI) = Ys/Yp

Relative stress index (RSI) = (Ys/Yp)/(Ȳs/Ȳp)

Mean productivity (MP) = (Yp + Ys)/2

Geometric mean productivity (GMP) = √(Ys × Yp)

Harmonic mean (HM) = 2(Ys × Yp)/(Ys + Yp)

In the formulas, Ys and Yp are yields of individual genotypes under stressed and control conditions, respectively, and Ȳs and Ȳp are the mean yields of all lines evaluated under the two contrasting conditions. For all indices, mean values of grain yield/plant (GYP, Table 1) were used. For the stress susceptibility index (SSI), considered one of the most useful indices to identify heat-tolerant genotypes (e.g., [24]), besides grain yield (GY1 and GYP), the grain number (GNS1 and GNP) and grain weight (TGW1 and TGW) parameters were also assessed, to estimate their relative contribution to the final yield in the different genotypes. TOL and SSI indices based on GYP figures are negatively correlated with yield under stress conditions (Ys); thus, lower values indicate higher stress tolerance (minimal reduction under stressful compared with non-stressful conditions). This rationale was followed to rank genotypes for SSI_GY and SSI_GN values, whereas for SSI_GW, the ability to increase it under stress was considered a positive attribute; therefore, genotypes were ranked in an opposite fashion. As for the other indices, relatively more tolerant genotypes are identified by higher values, being positively correlated with Ys and, some of them, with Yp as well (see [63,64,65]).

### 2.6. Statistical Analysis

One-way analysis of variance (ANOVA) was performed to estimate differences ascribable to the genotype (G) effect, while two-factor ANOVA was applied to analyse the effect of G × treatment (T) interaction. For those physiological parameters for which measurements were repeated over time (i.e., SPAD, SC, F_v_/F_m_, PI), a repeated-measures ANOVA was also performed. Whenever a significant F value was obtained for single factors or their interaction, the Tukey HSD test was performed at *p* < 0.05 level. Statistical analyses were performed by JASP software (JASP Team, 2022; JASP Version 0.16.1). For all parameters, two ANOVAs were carried out: in the first one, the datasets of each NIRL (+) and the corresponding non-carrier sib line (−) were compared to associate possible differences with the presence/absence of a given alien segment; in the second one, the datasets of the three NIRLs+ were compared to that of cv. Margherita.

## 3. Results

### 3.1. Intense Heat (IH) during Flowering: Main Traits Modulated

The majority of physiological, biochemical and yield-related traits assessed resulted highly significant for the G factor in both statistical comparisons (Table 2A,B), revealing strong constitutive differences among the tested genotypes. In the NIRLs+ vs. NIRLs− comparison, significant differences were observed for the G factor for all traits, except for stomatal conductance (SC), spike fertility index of the main culm (SFI1) and harvest index (Table 2A).

The heat treatment applied at anthesis (T factor) resulted to be highly impactful on most parameters, except for the Pro-SP trait, not affected by the IH stress imposition in both comparisons (Table 2A,B), and PH, showing no significant variation in the comparison between NIRLs+ and cv. Margherita (Table 2B). When the G × T interaction was considered, significant differences were observed between NIRLs+ and − in the variation of RWC, proline and sugar content (Pro-FL, Pro-SP, WSC-FL, WSC-SP) in stressed vs. unstressed conditions, indicating differential trait modulation between genotypes under the two tested conditions. These factors likely contributed to the significant G × T variation shown by grain yield (GY1, GYP) and to the highly significant variation of GNP (Table 2A). A higher number of statistically significant differences for the G × T interaction was observed in the comparison performed between NIRLs+ and Margherita, where, besides those mentioned above, other physiological (F_v_/F_m_, SC) and yield-related (GNS1, SFI and HI) parameters were also differently altered in the genotypes by the IH imposition (Table 2B).

### 3.2. Physiological Perturbations Occurring during IH Stress Application

**Figure 3:**
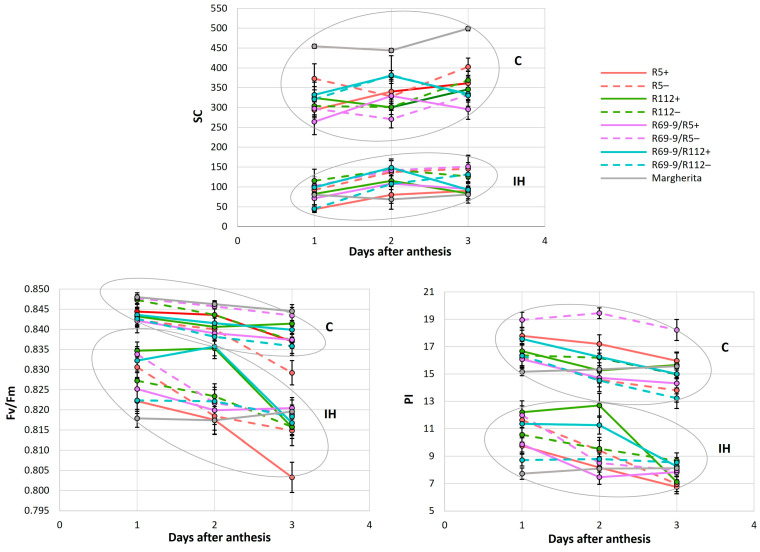
Mean values of stomatal conductance (SC), maximal photochemical efficiency of PSII (F_v_/F_m_) and Performance Index (PI) of unstressed (C) and intense-heat-stressed (IH) plants of NIRLs (+), their sib lines lacking the alien segment (−) and cv. Margherita at anthesis. For Tukey test values, see Table S1.

The results of the repeated-measures ANOVA (Table 3) and the subsequent Tukey HSD test for significant Time × G × T interactions (Table S1) showed that, among the NIRLs’ pairs (each NIRL+ vs. its sib−), the two parameters associated with chlorophyll fluorescence (F_v_/F_m_ and PI) were strongly influenced by both the timing of the measurements (i.e., stress duration) and the IH stress. All genotypes significantly decreased F_v_/F_m_ and PI under IH vs. the unstressed control (C) condition during the first or second day after the anthesis date, except for the recombinant genotypes R112+ and R69-9/R112+, in which F_v_/F_m_ remained almost unaltered, and PI was reduced to a lesser extent than in the other genotypes until day 2, before decreasing on the third day of stress application (Figure 3). This behaviour, which is indicative of more efficient PSII preservation under stress, is likely ascribable to the presence of the *Th. ponticum* segment shared by R112+ and R69-9/R112+ (Figure 1). Interestingly, even the heat-tolerant cv. Margherita had a significantly decreased F_v_/F_m_ value already after the first day of IH imposition (Figure 3 and Table S1B). As for SPAD (chlorophyll content) and SC (stomatal conductance), the observed differences that emerged from the repeated-measures ANOVA were not statistically significant (Table 3). Nonetheless, R112+ exhibited the lowest SPAD reduction on the third day of IH treatment (−1.86% vs. a mean of −7.34% in the other NIRLs+, Table S1A).

Regarding the SC trait, all the genotypes underwent a stomatal closure under the heat stress condition (Figure 3) ranging from 250–500 to 50–150 mmol/[m^2^s]. Interestingly, also Margherita, which showed the highest SC under C conditions, heavily reduced it (about −85%) in IH conditions, indicating the prompt stomata closure as a mechanism adopted by this tolerant genotype to cope with the severe heat stress applied at anthesis.

### 3.3. Membrane Damage Rate in IH-Stressed Leaves

The results obtained from the TBARS assay, performed on flag leaf tissues, revealed an expected general increase in lipid peroxidation levels after three days of IH stress imposition (Table S1). Even if ANOVA highlighted a statistically significant *p* value for both genotype and treatment factors for this trait (MDA-FL in Table 2), the interaction between them was not significant, indicating that all genotypes were similarly affected by the IH treatment (Table S1). Notwithstanding this, the lowest variations in MDA content in IH-stressed vs. control (C) plants were detected in R112+ (+6.7%), R112− (−6.4%) and R69-9/R5+ (+7.6%), the latter showing a conspicuously lower MDA content in IH condition compared with its corresponding sib−, i.e., R69-9/R5− (+47% in stressed vs. control plants), the lower damage being ascribable to the alien segment presence. By contrast, the IH stress caused substantial membrane damage to cv. Margherita, exhibiting an over 42% MDA increase vs. the C condition (Table S1B).

### 3.4. Osmolyte Accumulation in Flag Leaves and Spikes after Stress

**Figure 4:**
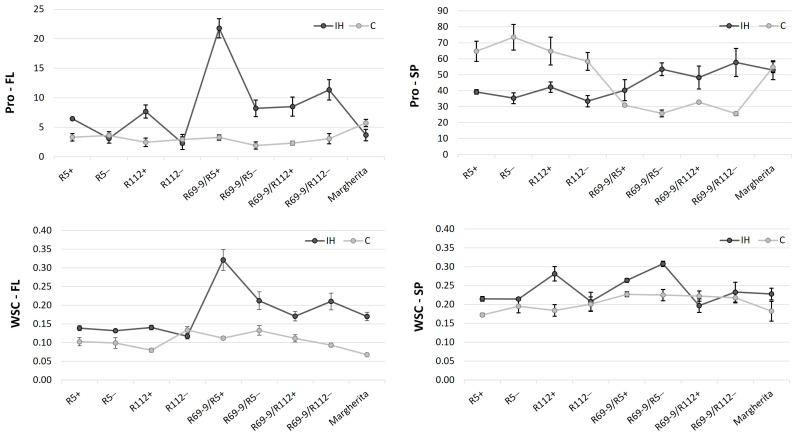
Proline (Pro) and water-soluble carbohydrate (WSC) content in control (C) and intense-heat-stressed (IH) flag leaves (FL) and spikes (SP) at anthesis. For Tukey test values, see Table S1A,B.

Changes in the total amounts of the osmolytes assayed, namely proline and water-soluble carbohydrates (WSC), were detected between the two conditions (control and heat stress) of the present experiment. The major variations due to stress application were found in FL tissues (Figure 4), with the recombinant line R69-9/R5+ emerging as the genotype that accumulates the highest levels of both osmolytes under the high-temperature regime. Since its corresponding sib− accumulated a statistically significant lower osmolyte content in FLs (Table S1A), this stress response mechanism seems to be under the control of the wild introgression present in R69-9/R5+. A positive trend could be also noticed for proline content in the FLs of R5+ and R112+, with the two NIRLs+ exhibiting higher proline accumulation than R5− and R112− after IH stress (Figure 4). A similar increment was not observed for WSC content, for which R5+ and R112+ showed comparable values to those of the corresponding sibs−. Thus, a specific effect on proline metabolism seems to be associated with *Th. ponticum* chromatin, although the boost observed in R69-9/R5+ probably benefited from positive interaction with factors controlled by its *Th. elongatum* 7EL segment.

As for osmolyte variation at the spike level, in all genotypes, proline accumulation in spikes was considerably higher than in FLs, regardless of the treatment applied. Among genotypes, the R69-9 types (*Th. ponticum* + *Th. elongatum*) possessed a lower proline amount under C conditions and increased it upon the IH application, not much differently from their sibs−. By contrast, the primary recombinants R5+ and R112+ (*Th. ponticum* only) showed a higher proline content in the C condition and a strong reduction, albeit not significant, in IH-stressed spikes (Table S1). On the other hand, WSC content in spikes was not strongly influenced by the IH stress, except for R112+, which displayed a 53% increase (IH vs. C conditions), whereas its corresponding sib− maintained its WSC content almost unchanged (Figure 4).

### 3.5. Relative Water Content Determination

**Figure 5:**
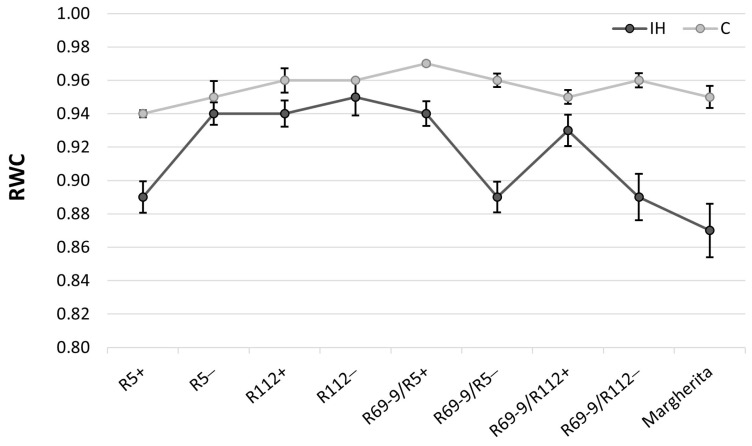
Relative water content (RWC) values in control (C) and intense-heat-stressed (IH) plants at anthesis. For Tukey test values, see Table S1A,B.

The relative water content (RWC) parameter, extensively used to determine the water status of the plant relative to the fully turgid condition, turned out to be highly modulated by the IH stress application (Figure 5). R5+ significantly reduced (−6%) RWC under IH vs. C conditions, compared with R5−, which maintained an almost unchanged RWC value (Table S1A). On the contrary, R69-9/R112+ exhibited a statistically higher RWC than R69-9/R112− under the IH condition (Figure 5, Table S1A), and a similar trend was true for the R69-9/R5+ vs. R69-9/R5− comparison, although the difference between these sib lines was not significant. The comparison between R112+ and its sib− line showed no difference between genotypes and conditions, indicating a positive effect of the common genetic background. On the other hand, cv. Margherita had the greatest RWC reduction of all genotypes upon stress application (−8.4% vs. C condition), despite its apparently efficient stomata closure (see above). Its RWC was significantly inferior to that of R112+, R69/R5+ and R69/R112+, while not statistically different from that of R5+ (Table S1B).

### 3.6. Effects of Heat Stress on Plant Productivity 

**Figure 6:**
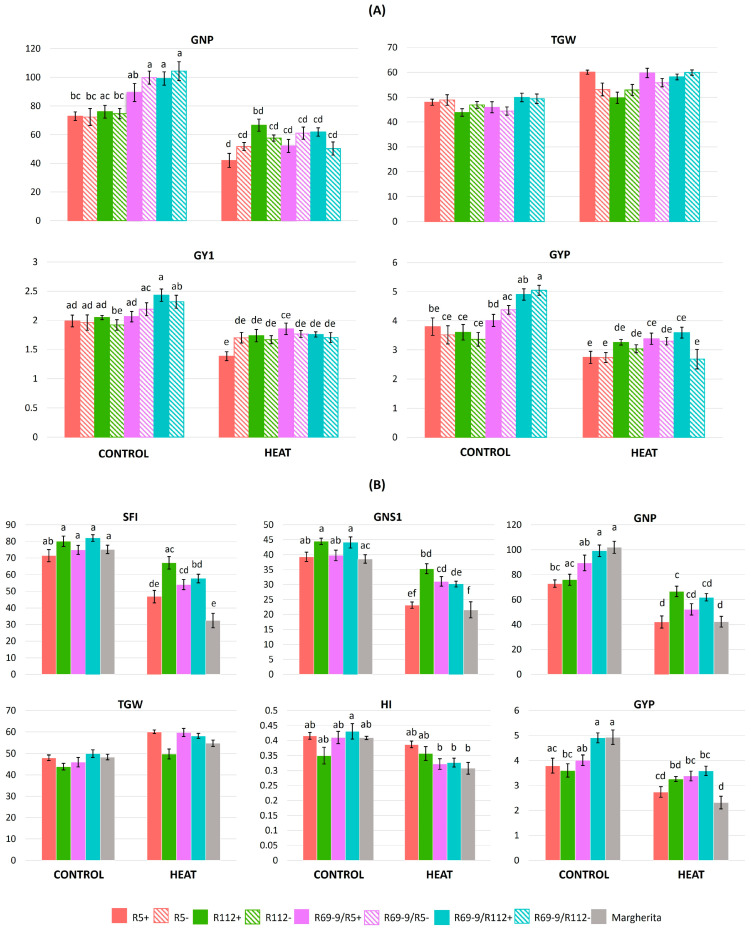
Effects of intense heat (IH) stress vs. control conditions on main yield-related traits: comparison between alien segment carrier (+) and non-carrier (−) NIRLs (**A**) and between NIRLs+ and cv. Margherita (**B**). Error bars represent standard errors of the means, and letters above histograms correspond to the ranking of Tukey test at *p* < 0.05 significance level. Reported traits showed significant G × T interaction, except for TGW (see Table 2). For trait acronyms, see Table 1.

As shown by ANOVA (Table 3), the intense heat treatment applied at anthesis had a severe impact on most yield traits. The most significant difference for the G × T interactions was associated with the GNP trait, followed by GYP and then by GY1 (Table 2). In fact, as a common response to the stress, all genotypes showed a reduction in grain number (GNS1, GNP; Figure 6), also evident in terms of the number of productive tillers/plant (PTP) compared with the total tiller number/plant (TNP) (Table S2), only partly compensated by an increase in grain weight (TGW1, TGW) and, as a result, a grain yield penalty (GY1, GYP). The impact on yield traits, however, varied greatly among genotypes. The minimum stress-induced reduction in spike fertility (−16% SFI) and grain number (−12% GNP) at the whole-plant level was exhibited by the primary recombinant R112+, which, with only a moderate increase in TGW (+13%), limited its yield loss to less than 10% at the whole-plant level and maintained its HI unchanged (Figure 6, Table S2A). A similar yield penalty was detected in R112−, which, however, had an almost doubled decrease in GNP (−23%) and HI reduced by 19.7%. By contrast, the heat stress caused a major decrease in GNP (−42%), PTP (−25%), GY1 (−30%) and GYP (−28%) parameters in the other primary recombinant, R5+, more conspicuous than in its R5− sib line (−28%, 0%, −13% and −22% for each of the three traits, respectively; Table S2A, Figure 6A). Consequently, R5+ lost the yield advantage over R5− displayed under normal conditions, despite a 25% increase in TGW (Table S2A, Figure 6A).

On the other hand, of the two secondary recombinants, it was R69/R5+ that better limited the effect of heat stress on yield compared with its sib− line and the R69/R112+ genotype: in R69/R5+, GY1 and GYP decreased by 10% and 15.7%, respectively, this genotype being apparently able to compensate the conspicuous reduction in GNP (−41.6%) with a considerable increase (+30%) in TGW. The R69/R5− line, lacking the alien introgression, was probably less capable of activating this trade-off mechanism, thus exhibiting a nearly 20% and 25% decrease in GY1 and GYP, respectively, in the stressed vs. control conditions (Table S2A, Figure 6A). The R69/R112+ NIRL was similarly less affected by the stress than its sib− line, although even in the former, GY1 and GYP were reduced by 25.6% and 26.8%, respectively (Table S2A, Figure 6A).

When all NIRLs+ were compared with Margherita (Table S2B), R112+, R69-9/R5+ and R69-9/R112+ were confirmed to possess statistically superior values under IH for yield traits such as the grain number (GNS1) and grain yield (GY1) of the main tiller and the spike fertility index of the whole plant (SFI). Moreover, the stressed plants of R112+ showed a significantly higher GNP than Margherita, substantiated by its highly stable SFI (−16% vs. −57%, respectively, under stress), with a similar impact on productive tillers (PTP). In terms of GYP, the stressed plants of R69-9/R5+ and R69-9/R112+ (and of R112+, though not in a significant manner) outperformed Margherita (Table S2B, Figure 6B). To this regard, it is noteworthy that in contrast with almost coinciding and top GYP values under normal conditions exhibited by R69-9/R112+ and Margherita (4.91 g and 4.94 g, respectively), the decrease observed as a result of the intense stress imposition was nearly doubled in the latter (−53%) compared with the former. This was largely due to a conspicuous reduction in spike fertility (−57% SFI) and grain number/plant (−58% GNP), not sufficiently compensated by a relatively modest grain weight increase (+13% TGW, Table S2B; see also Figure 6B).

The yield performance of the various genotypes under stressed vs. control conditions was also compared on the basis of stress indices. Besides the individual figures obtained by applying the respective mathematical formulas (see Materials and Methods), for each index, the nine tested genotypes were given a rank (from 1 to 9), and a mean of indices ranks (MIR) was calculated (Table 4). As a whole, the MIR confirmed the better performance (lower rank values) and hence the higher stress tolerance of R69-9/R5+ (MIR = 3), R112+ and R69-9/R112+ recombinant genotypes (in both MIR = 3.3) compared with their control (−) NIRLs and with the R5+ recombinant, as well as cv. Margherita (MIR = 6.7 and 7.8, respectively). However, the relative contribution of the different indices to the MIR of similarly tolerant genotypes, such as R112+, R69-9/R5+ and R69-9/R112+, varied. R112+, greatly supported by its background genotype, ranked first/second (tied with R112−) for TOL, SSI, YSI and RSI indices, which highlight yield stability under stress. For these indices, cv. Margherita had the lowest ranks (Table 4). On the other hand, R69-9/R112+ was the top-ranking genotype for MP, GMP, HM, YI and STI indices (with low ranks for the other ones), as frequently observed for genotypes with high yield potential under both stressed and unstressed conditions [63,64,65]. Good values for all stress indices (ranking 2 to 4, see Table 4) were detected for R69-9/R5+, which was thus confirmed to possess good yield stability under stress and high yield potential under stressful and favorable conditions.

A further test of genotype comparison was realised by calculating the stress susceptibility index (SSI) taking into consideration not only grain yield (both GYP, as in Table 4, and GY1, see Table 1 for acronyms) but also the main yield contributing traits, i.e., grain number (GN) and grain weight (GW), of both the whole plant (SSI_GNP and SSI_TGW, respectively) and the main (first) tiller (SSI_GN1 and SSI_TGW1, respectively). The results of Table 5 clearly show that the trait mainly contributing to the good yield stability of R112+ under stress was maintenance of GN, for which this NIRL ranked first, exceeding its NIRL− (R112−) both in SSI-GNP and SSI_GN1, thus representing an attribute evidently due to its alien segment introgression. In turn, for TGW, which was somewhat higher and more stable in R112− (see Table S2), the two NIRLs had a more similar ranking, as for the GY-based values (SSI_GYP and SSI_GY1, Table 5). On the other hand, quite opposite to R112+ behaviour was that of cv. Margherita, heavily penalised by the stress treatment in its potential to set seeds (lowest ranking in both SSI_GNP and SSI_GN1), to such an extent that, as above underlined, even its ability to develop large grains (see also [53]) could not avoid a major drop in final yield. Based on SSI values, R69-9/R5+ and R69-9/R112+ had a somewhat intermediate position, the former showing lower stress impact on GN traits, particularly at the main tiller level (SSI_GN1), the latter having a major support to final yield from GW, similarly more evident in SSI_TGW1 (Table 5).

## 4. Discussion

In the present study, the overall performance of primary DW-*Th. ponticum* near-isogenic recombinant lines (NIRLs), possessing slightly different amounts of the 7el_1_L chromosome arm replacing the distal end of the recipient DW 7AL arm, and of secondary DW-*Thinopyrum* spp. NIRLs, carrying a *Th. elongatum* 7EL chromosome segment distally “nested” into the 7el_1_L ones, was evaluated following the application of a severe day and night high-temperature regime during the highly sensitive and yield-critical stage of anthesis [10,20,66]. The specific treatment conditions adopted here (Figure 2) mimicked an intense, naturally occurring heat wave, i.e., a weather phenomenon when temperatures rise above the expected values for a continuous period of several days [67]. Moreover, stress imposition extended to night-time is known to worsen the negative effects on plant vitality and metabolic performance as compared with that applied at day or night only (see, e.g., [21]), greatly enhancing the energy cost (high respiration increase) required for growth and grain production [12,23]. Thus, the observed negative impact on most traits analysed was an expected outcome. Nonetheless, the results obtained here, based on a variation of physiological traits detected in flag leaves (FLs), osmolyte accumulation in yield-contributing tissues (FLs and spikes) and overall plant productivity, were useful in highlighting and substantiating the main mechanisms underlying the differential behaviours of the tested genotypes. As a result, the value of specific *Thinopyrum* spp. introgressions in enhancing DW tolerance towards a strong thermal stress emerged.

### 4.1. The Impact of Intense Heat Treatment on Flag Leaf Traits

As anticipated, all the physiological measurements performed on control and stressed plants confirmed that the heat regime applied caused an overall reduction in FLs’ chlorophyll content (SPAD), photosynthetic efficiency (F_v_/F_m_ and PI) and stomatal conductance (SC). Photosynthesis is known to be severely hampered by heat stress, mainly due to chloroplast disruption, inactivation of key enzymes, notably RuBisCO, and inhibition of PSII [16,68,69,70]. Closely connected with the maintenance of the photosynthetic process is the rate of CO_2_ uptake and hence the regulation of stomatal aperture [71]. In fact, stomatal opening is generally induced by heat stress to promote evaporative cooling [72,73,74]. Here, stomata closure was a stress response mechanism adopted by all genotypes, with no significant difference among them (Figure 3; Table S1). This observation is in line with data previously recorded on some of the genotypes tested in the present work (R5 and R112 NIRLs) but subjected to less intense heat stress conditions [53] and with other studies reporting a decreased SC in well-watered plants exposed to heat phenomena [26,75]. Stomatal closure to prevent the decline in leaf water potential and avoid dehydration characterizes an isohydric behaviour of species/genotypes that, in facing heat waves, have larger hydraulic safety margins, thanks to a variety of strategies and features, including a well-developed and deep rooting system [17,24,26,76]. Interestingly, a previous analysis of the seminal root architecture of DW-*Th. ponticum* recombinants [77] showed the 5% 7el_1_L segment differentiating the R112+ from R5+ NIRLs (see Figure 1) to determine a significant increase in several root traits, such as spread of root angle, average root diameter, biomass and length. In the present study, the R112+ line exhibited the lowest stress-induced SPAD decrease among the NIRLs+ after 3 days of stress imposition (Table S1), similar to what has been previously observed at anthesis and post-anthesis stages (grain filling) in various stressful field environments [48,49]. In the same trials, the remarkable ability displayed by R112+ to maintain prolonged greenness was also supported by other photosynthetic parameters measured on FLs, i.e., nitrogen balance index and flavonol content [48]. In the present investigation, both R112+ and its R69/R112+ derivative showed a distinctive capacity to retain high photosynthetic efficiency during the first 2 days of stress (Figure 3, Table S1). Since the same behaviour was not observed in the corresponding sibs−, nor in any other of the analysed genotypes, including cv. Margherita, the preservation of the overall photosynthetic performance appears to be specifically associated with the presence of the proximal portion of the *Th. ponticum* segment shared by R112+ and R69-9/R112+ (Figure 1). This evidence of photosynthetic stability under intense heat stress at anthesis was detected here for the first time and, combined with previous field data recalled above, supports the hypothesis of representing one of the underlying mechanisms of the improved heat tolerance observed for these two genotypes, particularly R112+, also in terms of yield stability. In fact, selection for heat-tolerant plants based on high F_v_/F_m_ values is a widely used strategy [78,79]. Zhang et al. [80] reported that bread wheat cultivars exposed to a 35/25 °C d/n temperature regime showed a strong PSII photoinhibition, with a significant reduction in F_v_/F_m_ even 1 day after the heat stress. In our case, a wide difference was evident among the tested materials: apart from the R112+ and R69-9/R112+ cases, photosynthetic efficiency was impaired to a similar extent in all other genotypes during the three days of stress duration. In this respect, Margherita was no exception, displaying a nearly 50% reduction in PI on each of the three days of stress, despite maintaining a constantly high chlorophyll content over the stress duration (Table S1B).

Confirming the negative impact that the intense heat stress had on FL tissue, an overall increase in MDA content, produced when polyunsaturated fatty acids in the membrane undergo peroxidation, was detected in almost all genotypes (Table S1). This outcome is in line with similar evidence from other studies, where increased lipid peroxidation, being part of the oxidative damage, was found in wheat genotypes as a consequence of intense thermal stress [81,82,83]. In the present investigation, despite no significance in MDA variation among the tested lines, the percentage difference of certain genotypes under stressed vs. control conditions, notably of R112+, R69-9/R5+ and R69-9/R112+ on one hand and cv. Margherita on the other, appears noteworthy (Table S1B). The conspicuous MDA increase in the latter substantiates the greatly reduced PI (see above), being likely correlated with the functional impairment of PSII in chloroplast lamellae (see, e.g., [81]).

Still at the FL level, the chromosomal and genetic makeup shared by R69-9/R5+ and R69-9/R112+ (Figure 1) positively influenced RWC preservation under stress, since these two recombinants did not significantly decrease their RWC under IH vs. C conditions, whereas the corresponding lines lacking the wild chromatin (NIRLs−) did (Figure 5; Table S1A). RWC was also maintained in R112+, as was in a previous study under less severe heat stress [53], although in the present analysis, its behaviour overlapped with that of its NIRLs− (Figure 5). By contrast, with increasing heat stress severity, the R5+ line and cv. Margherita were apparently unable to save water in their leaf tissues (see also the overall photosynthetic penalty shown by these genotypes, Figure 3 and Table S1), the stomata closure mechanism being evidently insufficient to assure the maintenance of tissue hydration and correlated physiological activities.

### 4.2. The Role of Osmolytes in Tolerance to Intense Heat

The particularly positive stress adaptation and tolerance exerted by R69-9/R5+ (top ranking in the mean of stress indices, Table 4) might have also been due to the remarkable increase in proline and soluble sugars (WSC) detected in FLs at the end of the 3 days of IH stress application. In fact, R69-9/R5+ exhibited the highest osmolyte content in FLs under stress, a general response adopted by R69-9 NIRLs (both “+” and “−”), though to a minor degree compared with R69-9/R5+ (Figure 4). Generally, osmolyte compounds accumulate in plants under several stress conditions, and their high concentration is linked to superior tolerance [84,85,86]. This is in agreement with several studies, in which stress-tolerant (including heat-tolerant) wheat plants challenged at anthesis and post-anthesis stages were shown to accumulate high amounts of osmoprotectants in leaves, thus ameliorating the stress response through enhanced osmotic adjustment, RWC maintenance and membrane stability (e.g., [27,29,87,88,89,90]). As for WSC, their accumulation in leaves and other organs (see ahead) under abiotic stress (e.g., [29,91]) has a likely dual significance, i.e., directly linked to photosynthetic activity as well as to their osmoprotective function. This is widely recognised for leaf blades, especially of FLs during late developmental stages, and more recently emphasised also for non-foliar organs, such as peduncles and spikes. Spike organs, in particular, due to their special morphological, physiological and metabolic features and inherent delayed senescence, are more heat- and water-deficit stable than FLs and represent the main photosynthetic contributors during grain filling ([31,91,92] and references therein). Interestingly, post-heading high night-time temperature exposure was shown to induce significantly higher metabolic changes in the wheat spike compared with the leaf and stem [88]. In that context, higher levels of soluble sugars in spikes of the tolerant genotype were interpreted as indicative of increased starch breakdown to meet the increased respiratory demand, with some sugars, like raffinose and maltose, acting as key osmoprotectants ([88] and references therein).

In the present work, osmolyte contents in spikes as compared with FLs were largely superior for proline, as previously observed under milder temperature stress to which some of the present materials (R5 and R112 NIRLs) were exposed [53]. However, variation among genotypes seemed to be more correlated to the genealogy of the NIRLs (primary vs. secondary types) than to the presence/absence of a specific alien chromosome segment (Figure 4 and Table S1). On the other hand, WSC content was of similar magnitude in the two organs and across genotypes, except for R112+, the only one that showed a significant increase at the spike level (+53%, Table S1A). The same increase was not previously observed in this NIRL and other genotypes under less severe heat stress [53], suggesting the response to be associated with stress intensity and duration. Dreccer et al. [93] showed bread wheat recombinant inbred lines with contrasting WSC in stems and sheaths at anthesis to differ significantly in carbon (C) metabolism, spike biomass development and productive performance when subjected to higher (28/14 °C d/n) than normal (20/10 °C) temperatures from terminal spikelet until anthesis stage. Their results supported a model where higher biomass in the spike (e.g., by higher partitioning or C fixation in high-WSC lines), contributed to a higher amount of glucose in the spike, and this, in turn, contributed to a higher number of fertile florets. In the same study, the grain set was also at an advantage for high- vs. low-WSC lines.

### 4.3. Genotypes’ Stress Response for Yield Traits

The outline emerging from the work of Dreccer et al. [93] appears to apply rather well to the R112+ genotype, which combines high WSC content in stressed spikes with great stability of spike biomass and fertility compared with the other recombinant lines and cv. Margherita (see the SFI1 and SFI traits in Table S2B). Concomitantly, R112+ had the smallest decrease in grain number following the IH treatment, both at the spike (GN1) and at the plant (GNP) level (Table S2B). The superiority of R112+ for grain-number parameters detected here reinforces previous observations across different and contrasting environments, under both field [48,49] and controlled conditions of moderate heat stress [53].

A major impact on grain-number-related traits was observed in bread wheat subjected to heat stress of similar intensity and duration (5 days-lasting 36/26 °C d/n temperature) to those of the present investigation [94]. In that study, the same treatment was imposed at many defined times across anthesis (from 15 d before to 30 d after), highlighting how the significant yield losses caused by the high temperature were maximum in two periods, 8–6 d and 2–0 d before anthesis, and corresponded with a decrease in floret fertility and seed set, largely due to loss of pollen fertility and abnormalities in pollen, stigma and style (see also [12,88]). Significantly decreased grain weight (GW) was instead detected when stress episodes occurred 10–30 days after anthesis, i.e., at the grain filling stage. In the same study [94], GW increase as a compensatory mechanism for decreased spike fertility due to an earlier stress, with more assimilates available to the extant developing grains, was found to be of minor entity.

Here, genotypes performed differently in the trade-off between GN and GW under heat stress (see Table S2; see also [67]). The GW contribution, although the G × T interaction was not statistically significant for this trait (Table 2; Table S2), was minimal in R112+, thanks to the important contribution to the yield stability of its GN attributes (<10% of yield penalty under stress), as above underlined. A similar trend was observed for the R112-derived secondary recombinant, R69-9/R112+, although its GW increments did not satisfactorily compensate for the higher GN decrease compared with R112+, which overall resulted in nearly 27% yield penalty (Table S2). Stressed plants of the R5+ NIRL were able to support a conspicuous GW increase in set seeds, which seems to be a distinctive feature conferred by its *Th. ponticum* segment under natural and imposed stress conditions [49,53]. Nonetheless, under the stress conditions imposed here, this was not sufficient to offset the GN drop in final yield formation. On the other hand, an even higher GW increase, likely sustained by an efficient physiology of source organs (RWC, Pro-FL, WSC-FL, see above), enabled R69-9/R5+ plants to limit yield loss to <16%, despite the heat-induced reduction in GN trait values (Table S2). In fact, the genotypes’ appraisal based on various stress susceptibility indices, which take into account yield potential under favourable conditions and yield under stress, here indicated R69-9/R5+ as the top-ranking one (Table 4).

Meaningful support for the outstanding ability to tolerate prolonged heat stress accompanying critical growth stages comes from consolidated evidence from multi-year field trials carried out in Biskra (Algeria), a very hot and arid environment located south of the Saharan Atlas. There, with temperatures reaching 15/24 °C (min) and 27/30 °C (max) at flowering (mid-April) and 20–35 °C (min–max) at grain filling (early May), combined with very low air humidity, irrigation is an indispensable measure to obtain any harvest. Under localised plot irrigation management, the R69-9/R5+ recombinant line was consistently the top-ranking one among other DW-*Thinopyrum* spp. lines, as well as several DW traditional and elite varieties from various countries surrounding the Mediterranean basin ([51] and unpublished). Whereas part of the excellent performance of R69-9/R5+ can be ascribed to the great salinity tolerance conferred by its *Th. elongatum* introgression into the *Th. ponticum* segment (Figure 1; see also [52]) in the highly saline Biskra environment, the ability to withstand the extreme heat (in the absence of water deficit in the specific trials) was an additional important asset. Under those stressful conditions, R69-9/R5+ owed its high yield to appreciable values of all main yield contributing traits (from TNP to GN/spike and TGW), a good balance that the line was evidently able to maintain also under the particularly stressful conditions of the present experiment.

By contrast, the same confirmation cannot be given for cv. Margherita, which not only showed good performance in Biskra (particularly for TGW and final yield) but also a quite good response in terms of grain production when heat shocks were imposed during daytime at anthesis, alone and in combination with water deficit [53]. The choice of cv. Margherita as a heat-tolerant reference was based on its good performance across various stressful environments, although with some exceptions [55,56,95]. Notably, Margherita resulted within the one-third top yielders in irrigated field trials carried out in a locality of the Senegal River basin (Fanaye, Senegal) characterised by extremely high daytime temperatures at anthesis (34/37 °C), but where night temperatures average 16 °C [55]. One of the possible reasons for the contrasting behaviour shown by Margherita under the stress conditions applied in the present investigation could lie precisely in the strikingly different night temperatures. Here, the intense heat stress extended to night-time probably caused a series of perturbations (from enhanced respiration rates to an increase in ROS, with consequent cell damage, decreased pollen viability and floret fertility, to mention some; see also above and [12,19,22,23,88]) that profoundly upset the metabolic/energetic/physiological machinery available to this variety, preventing it from adequately investing in crop formation. The same could apply to the R5+ case, also displaying lower performance than previously observed under stressful field environments (e.g., [49]) and controlled conditions [53].

## 5. Conclusions

The heat treatment conditions of the present experiment were rather extreme, both at daytime and night-time. To give a reference figure, a drop of 3 °C only in maximum day temperature at flowering time in Fanaye, Senegal (from 37 °C in 2016 to 34 °C in 2017), pushed the average GY to nearly double that achieved in the hotter season [55]. Furthermore, the application of high night-time temperature, whose greatly detrimental effects have been underlined above, apparently represented a critical discriminating factor among the tested genotypes to highlight the best responding and the most distinctive adaptive and tolerance mechanisms they adopted. 

Our work demonstrates how the presence of small, targeted introgressions of *Thinopyrum* spp. chromatin, developed through a precise and sustainable “chromosome engineering” approach, allows endowing durum wheat with tolerance to intense heat when imposed throughout day and night at the sensitive stage of flowering, besides additional responsive attributes to a variety of biotic and abiotic stresses (see Introduction). The accurate phenotyping we carried out enabled the identification of the most interesting and breeding-attractive genotypes among the tested DW-*Thinopyrum* spp. recombinant lines. This is the case of R112+, possessing a 28%-long *Th. ponticum* 7el_1_L segment, which stood out for its remarkable yield stability under stress. The maintenance of RWC and photosynthetic efficiency, as well as the enhanced sugar content in the spike, appear as major physiological mechanisms underlying its yield performance, characterised by high spike fertility and hence high grain number. Notably, this is widely considered the single most appealing target trait for breeding better heat tolerance (e.g., [55] and references therein). Among the secondary recombinants, here tested for the first time under the specific stress conditions, particularly noteworthy was the tolerance exhibited by R69-9/R5+. Its *Th. elongatum* 7EL chromosome segment proved to majorly contribute to yield preservation through RWC maintenance and a superior proline and WSC accumulation in flag leaves under stress. Thus, it would be ideal to use such excelling recombinant genotypes as parents in crosses with adapted, high-yielding wheat varieties (of both durum and bread wheat), in which their specific alien features could be profitably incorporated and exploited to enhance yield and yield stability in view of increasingly intense and threatening weather events and climate change.

## Figures and Tables

**Table 1:** Traits assessed and corresponding acronyms adopted.

Measurement/Analysis	Acronym
Chlorophyll content (SPAD units)	SPAD
Max photochemical efficiency of PSII	F_v_/F_m_
Performance Index	PI
Stomatal conductance (mmol/[m^2^s])	SC
Relative water content	RWC
Flag leaf proline content (µmol/g FW)	Pro-FL
Spike proline content (µmol/g FW)	Pro-SP
Flag leaf water-soluble carbohydrate content (g/g DW)	WSC-FL
Spike water-soluble carbohydrate content (g/g DW)	WSC-SP
Flag leaf malondialdehyde content (µmol/g FW)	MDA-FL
Grain number/spike (main tiller)	GNS1
Spike fertility index (main tiller)	SFI1
Thousand grain weight (main tiller, g)	TGW1
Grain yield (main tiller, g)	GY1
Plant height (cm)	PH
Tiller number/plant	TNP
Productive tiller number/plant	PTP
Grain number/plant	GNP
Spike fertility index	SFI
Thousand grain weight (g)	TGW
Grain yield/plant	GYP
Harvest index	HI

**Table 2:** ANOVA probability (*p*) values for physiological, biochemical and yield-related traits measured under stressed and control conditions: (**A**) statistical comparison between NIRL+ and corresponding sib− genotypes; (**B**) statistical comparison between NIRLs+ and cv. Margherita. *, ** and *** indicate significance at *p* < 0.05, *p* < 0.01 and *p* < 0.001, respectively (df = degrees of freedom; G = genotype, T = treatment). For trait acronyms, see Table 1.

A				B			
Factors	G	T	G × T	Factors	G	T	G × T
df	7	1	7	df	4	1	4
TRAITS	*p* Values			TRAITS	*p* Values		
SPAD	<0.001 ***	0.002 **	0.13	SPAD	<0.001 ***	0.019 *	0.484
Fv/Fm	<0.001 ***	<0.001 ***	0.193	Fv/Fm	0.015 *	<0.001 ***	0.013 *
PI	<0.001 ***	<0.001 ***	0.075	PI	0.008 **	<0.001 ***	0.219
SC	0.159	<0.001 ***	0.517	SC	<0.001 ***	<0.001 ***	<0.001 ***
RWC	<0.001 ***	<0.001 ***	0.002 **	RWC	<0.001 ***	<0.001 ***	0.016 *
Pro-FL	<0.001 ***	<0.001 ***	<0.001 ***	Pro-FL	<0.001 ***	<0.001 ***	<0.001 ***
Pro-SP	0.044 *	0.33	<0.001 ***	Pro-SP	0.031 *	0.248	0.014 *
WSC-FL	<0.001 ***	<0.001 ***	<0.001 ***	WSC-FL	<0.001 ***	<0.001 ***	<0.001 ***
WSC-SP	0.011 *	<0.001 ***	0.048 *	WSC-SP	0.018 *	<0.001 ***	0.011 *
MDA-FL	0.003 **	<0.001 ***	0.181	MDA-FL	0.014 *	0.006 **	0.48
GNS1	<0.001 ***	<0.001 ***	0.165	GNS1	<0.001 ***	<0.001 ***	0.048 *
SFI1	0.128	<0.001 ***	0.51	SFI1	0.303	<0.001 ***	0.589
TGW1	<0.001 ***	<0.001 ***	0.481	TGW1	<0.001 ***	<0.001 ***	0.32
GY1	<0.001 ***	<0.001 ***	0.044 *	GY1	<0.001 ***	<0.001 ***	0.008 **
PH	<0.001 ***	<0.001 ***	0.164	PH	<0.001 ***	0.071	0.163
TNP	<0.001 ***	<0.001 ***	0.279	TNP	0.340	<0.001 ***	0.761
PTP	0.194	0.804	0.498	PTP	0.011 *	0.618	0.369
GNP	<0.001 ***	<0.001 ***	<0.001 ***	GNP	<0.001 ***	<0.001 ***	<0.001 ***
SFI	<0.001 ***	<0.001 ***	0.104	SFI	<0.001 ***	<0.001 ***	<0.001 ***
TGW	<0.001 ***	<0.001 ***	0.146	TGW	0.001 **	<0.001 ***	0.104
GYP	<0.001 ***	<0.001 ***	0.002 **	GYP	<0.001 ***	<0.001 ***	<0.001 ***
HI	0.106	<0.001 ***	0.268	HI	0.314	<0.001 ***	0.05 *

**Table 3:** Repeated-measures ANOVA probability (*p*) values for physiological measurements performed over the three days of IH stress application for Time, Time × Genotype, Time × Treatment and Time × Genotype × Treatment factors. (**A**) Statistical comparison between NIRLs+ and corresponding sibs−. (**B**) Statistical comparison between NIRLs+ and Margherita. *, ** and *** indicate significance at *p* < 0.05, *p* < 0.01 and *p* < 0.001, respectively (df = degrees of freedom; G = genotype, T = treatment). For trait acronyms, see Table 1.

A					B				
Factors	Time	Time × G	Time × T	Time × G × T	Factors	Time	Time × G	Time × T	Time × G × T
df	2	14	2	14	df	2	8	2	8
TRAITS	*p* Values				TRAITS	*p* Values			
SPAD	0.251	0.991	0.229	0.991	SPAD	0.363	0.997	0.355	0.942
Fv/Fm	<0.001 ***	0.037 *	0.105	0.043 *	Fv/Fm	<0.001 ***	0.044 *	0.077	0.047 *
PI	<0.001 ***	0.345	0.513	0.019 *	PI	<0.001 ***	0.013 *	0.584	0.188
SC	0.002 **	0.74	0.305	0.457	SC	0.177	0.442	0.752	0.736

**Table 4:** Values of stress tolerance indices calculated on the basis of yield/plant of each genotype when grown under normal (Yp) and stressed (Ys) conditions. Values were ranked (1 to 9) and colour-coded based on Excel scales (colour gradient from intense green = best values to intense red = worst values); the same colour gradient was applied to means of indices ranks (MIR) determined for each genotype. See Section 2 for acronyms, formulas and ranking rationale.

Genotype	Yp	Ys	Stress Tolerance Indices																		
			TOL		MP		GMP		HM		SSI		STI		YI		YSI		RSI		MIR
			Value	Rank	Value	Rank	Value	Rank	Value	Rank	Value	Rank	Value	Rank	Value	Rank	Value	Rank	Value	Rank	
R5+	3.80	2.74	1.06	5	3.27	7	3.23	7	3.18	7	0.99	7	0.60	7	0.91	6	0.72	7	1.00	7	6.67
R5−	3.52	2.74	0.78	4	3.13	9	3.11	9	3.08	9	0.79	4	0.55	9	0.91	6	0.78	4	1.08	4	6.44
R112+	3.61	3.26	0.35	2	3.44	6	3.43	5	3.43	5	0.34	1	0.67	5	1.08	4	0.903	1	1.255	1	3.33
R112−	3.37	3.04	0.33	1	3.21	8	3.20	8	3.20	6	0.35	2	0.59	8	1.01	5	0.902	2	1.254	2	4.67
R69-9/R5+	4.01	3.38	0.63	3	3.70	4	3.68	3	3.67	3	0.56	3	0.78	3	1.12	2	0.84	3	1.17	3	3.00
R69-9/R5−	4.38	3.30	1.08	6	3.84	3	3.80	2	3.76	2	0.88	5	0.83	2	1.10	3	0.75	5	1.05	5	3.67
R69-9/R112+	4.91	3.59	1.32	7	4.25	1	4.20	1	4.15	1	0.96	6	1.01	1	1.19	1	0.73	6	1.02	6	3.33
R69-9/R112−	5.05	2.68	2.37	8	3.87	2	3.68	4	3.50	4	1.67	8	0.78	4	0.89	8	0.53	8	0.74	8	6.00
Margherita	4.94	2.32	2.62	9	3.63	5	3.39	6	3.16	8	1.89	9	0.66	6	0.77	9	0.47	9	0.65	9	7.78

**Table 5:** Stress susceptibility index (SSI) values calculated considering GY and its components (GN and TGW) for the whole plant and its main tiller. Values were ranked (1 to 9) and colour-coded based on Excel scales (colour gradient from intense green = best values, to intense red = worst values). See Section 2 for acronyms, formulas and ranking rationale.

Genotype	SSI—Plant Values						SSI—1st Tiller Values					
	GYP	Rank	GNP	Rank	TGW	Rank	GY1	Rank	GN1	Rank	TGW1	Rank
R5+	0.99	7	1.10	7	1.36	3	1.39	8	1.42	8	1.59	2
R5−	0.79	4	0.74	3	0.46	9	0.61	3	0.77	3	1.60	1
R112+	0.34	1	0.32	1	0.73	6	0.69	4	0.71	1	1.09	4
R112−	0.35	2	0.59	2	0.70	8	0.60	2	0.80	4	0.80	6
R69-9/R5+	0.56	3	1.08	6	1.63	1	0.46	1	0.76	2	1.32	3
R69-9/R5−	0.88	5	1.01	5	1.39	2	0.89	5	0.89	5	0.85	5
R69-9/R112+	0.96	6	0.98	4	0.89	5	1.26	7	1.09	7	0.52	9
R69-9/R112−	1.67	8	1.34	8	1.16	4	1.21	6	1.07	6	0.66	8
Margherita	1.89	9	1.52	9	0.72	7	1.84	9	1.53	9	0.68	7

## Data Availability

The original contributions presented in this study are included in the article/Supplementary Materials, further inquiries can be directed to the corresponding authors.

## Supplementary Materials

The following supporting information can be downloaded at https://www.mdpi.com/article/10.3390/plants13182605/s1, Table S1: Physiological traits’ means, standard errors (SE), ranking of Tukey test at *p* < 0.05 level in each column and percentage (%) difference of mean values for each genotype between stress (IH) and control (C) conditions. Table S2: Yield traits’ means, standard errors (SE), ranking of Tukey test at *p* < 0.05 level in each column and percentage (%) difference of mean values for each genotype between stress (IH) and control (C) conditions.
